# Malingering

**Published:** 1890-02-01

**Authors:** 


### MALINGERING.

In a former "Retrospect" we referred to a case of malingering, and now note a second. Mr. Nunn, consulting surgeon Middlesex Hospital, details a strange case of malingering (*Lancet*, January 4th). A girl æt. sixteen had sores on the leg; there were also patches of skin which had apparently lost vitality, but obviously not slough. Sores and patches were curiously quadrangular in shape. On the arms and chest were cicatrices. Two squares of blue soap were found in the patient's possession and removed as not included in the list of patients' necessaries. The sizes of the sores and the soap coincided. Of course when the soap was taken away no more sores appeared. After keeping the soap in a dry place a hard crystalline incrustation of strong alkali formed on the surface. Mr. Nunn expresses surprise that one so young could have discovered that binding a strongly alkaline soap to the skin would produce sores. She must have done so for some time before she was sixteen, as the cicatrices on the chest were not recent. Malingering in one so young, which involves much physical suffering, is rare, although common enough in older people, particularly among some classes of soldiers, especially where enlistment is not voluntary.

